# Supplementary material for: Comprehensive Analysis Reveals That ISCA1 Is Correlated with Ferroptosis-Related Genes Across Cancers and Is a Biomarker in Thyroid Carcinoma
Source: Genes (Basel). 2024 Nov 28;15(12):1538. doi: 10.3390/genes15121538 (PMC11675480; doi:10.3390/genes15121538)
Supplement: Supplementary file 1 [file genes-15-01538-s001.zip › Supplementary Figures and Tables.pdf]

Supplementary Figures and Tables

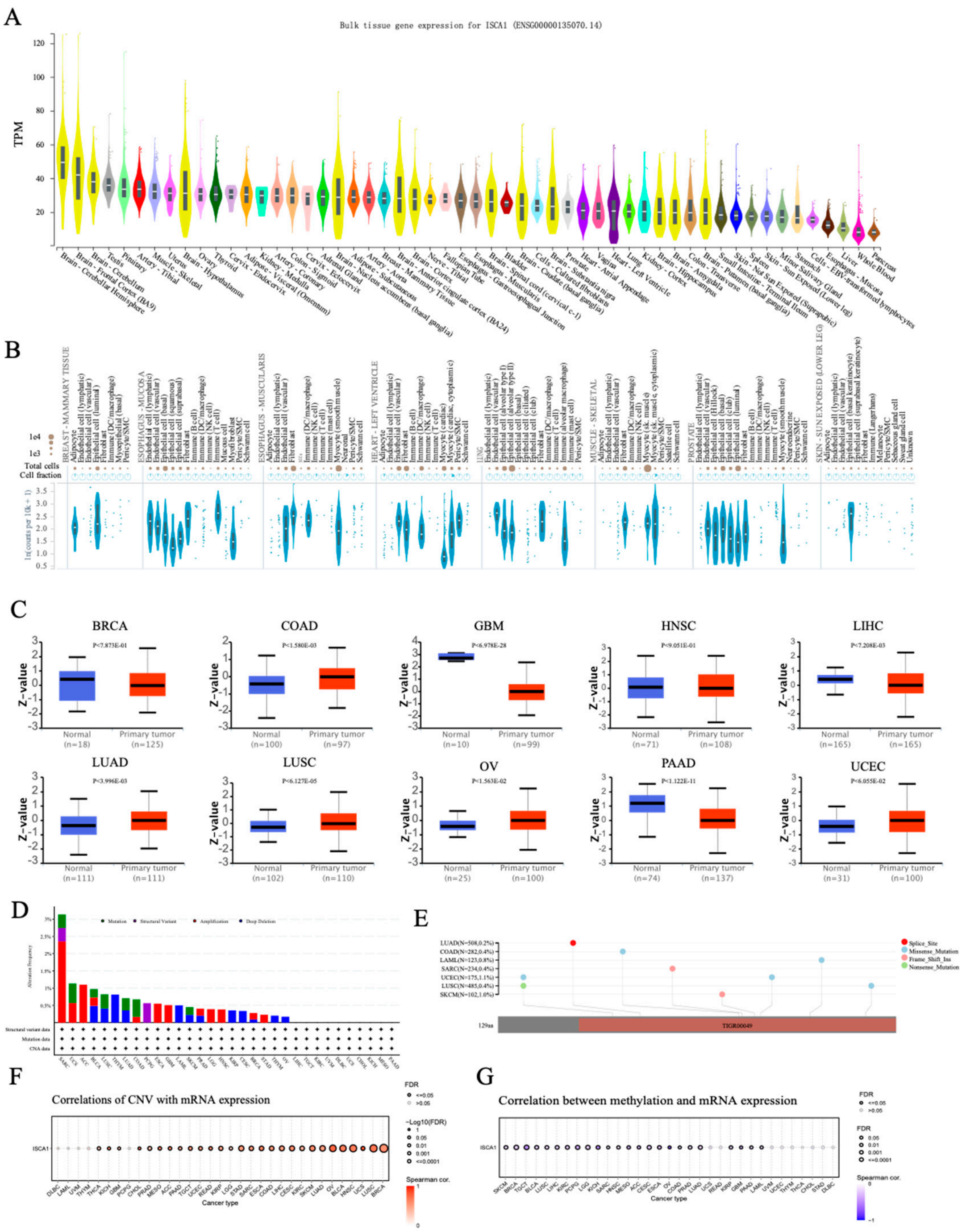

Supplementary Figure S1. The expression of ISCA1 in pan cancer. (A) Violin plots showing ISCA1 expression levels in various human normal tissues. **(B)** Violin plots displaying the single-cell expression data of ISCA1 in human normal breast, esophagus, heart, lung, muscle, prostate and skin tissues. **(C)** Representative images of immunohistochemical staining of ISCA1 in 3 types of normal and tumor tissues. **(D)** Alteration frequency of ISCA1 in various tumors. **(E)** Mutation types and chromosomal location of ISCA1 in various tumors. **(F)**

Association between CNV and mRNA expression. **(G)** Association between Methylation and mRNA expression.

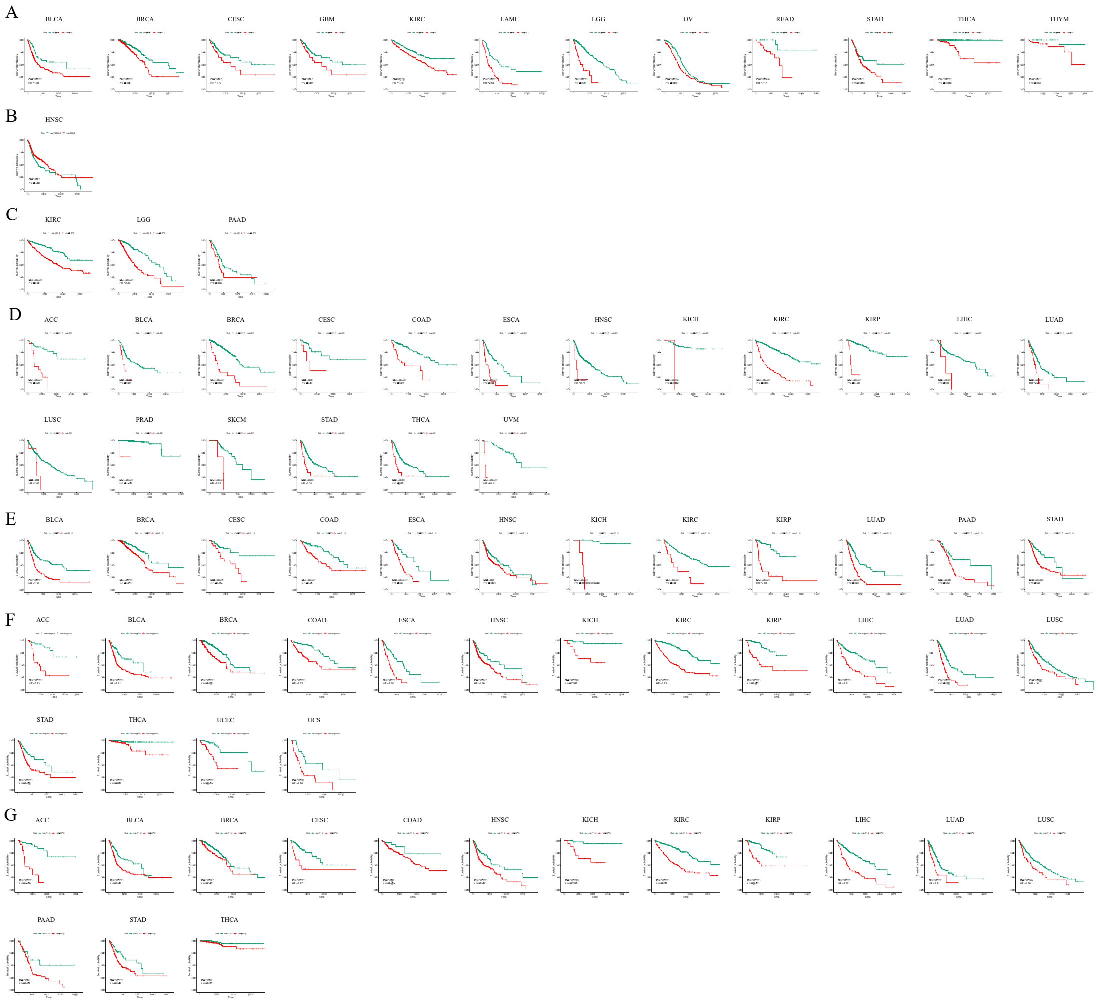

Supplementary Figure S2. Predictive value of *ISCA1* expression in overall survival (OS) in pan-cancer.

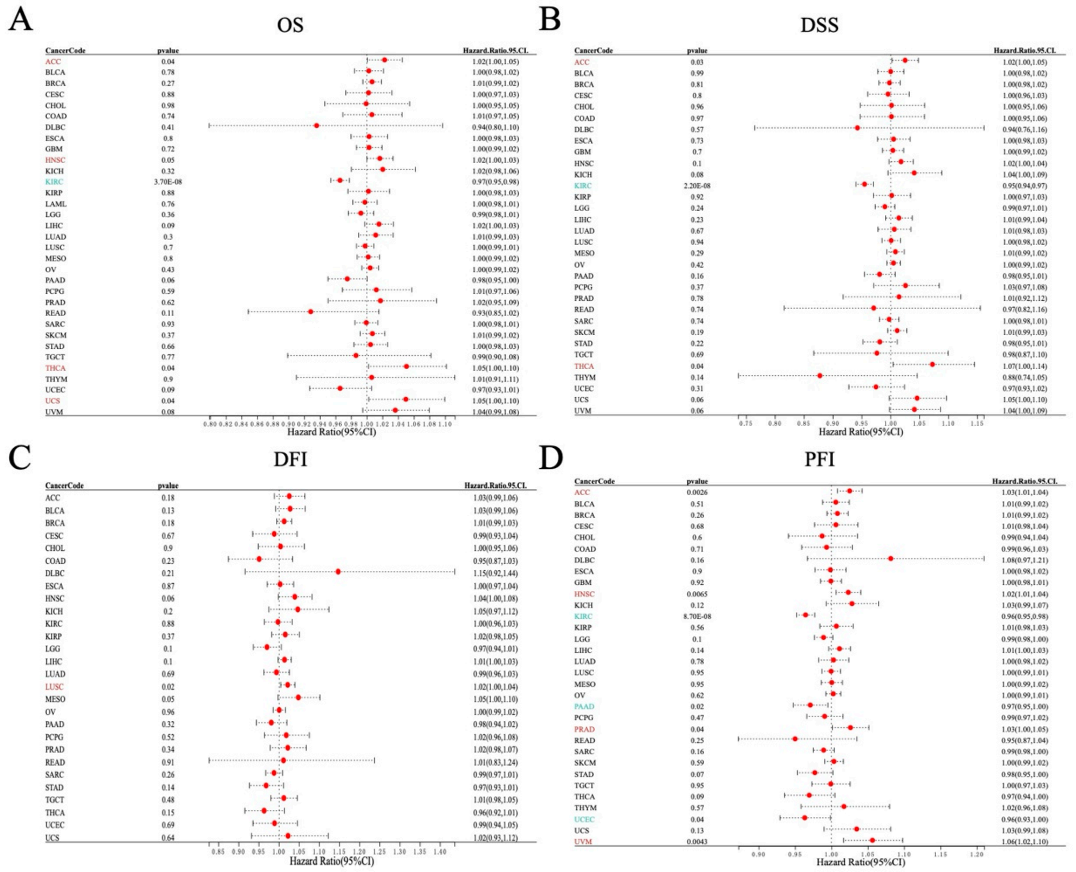

Supplementary Figure S3. Forest plot displays the survival of ISCA1 RNA expression levels in pan cancer.

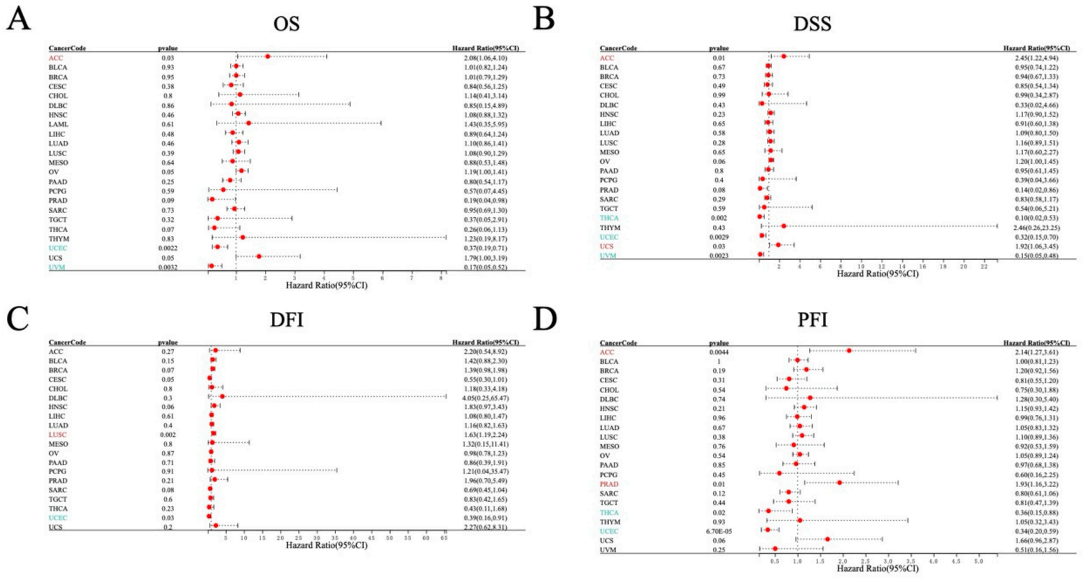

Supplementary Figure S4. Forest plot displays the survival of ISCA1 CNV expression levels in pan cancer.

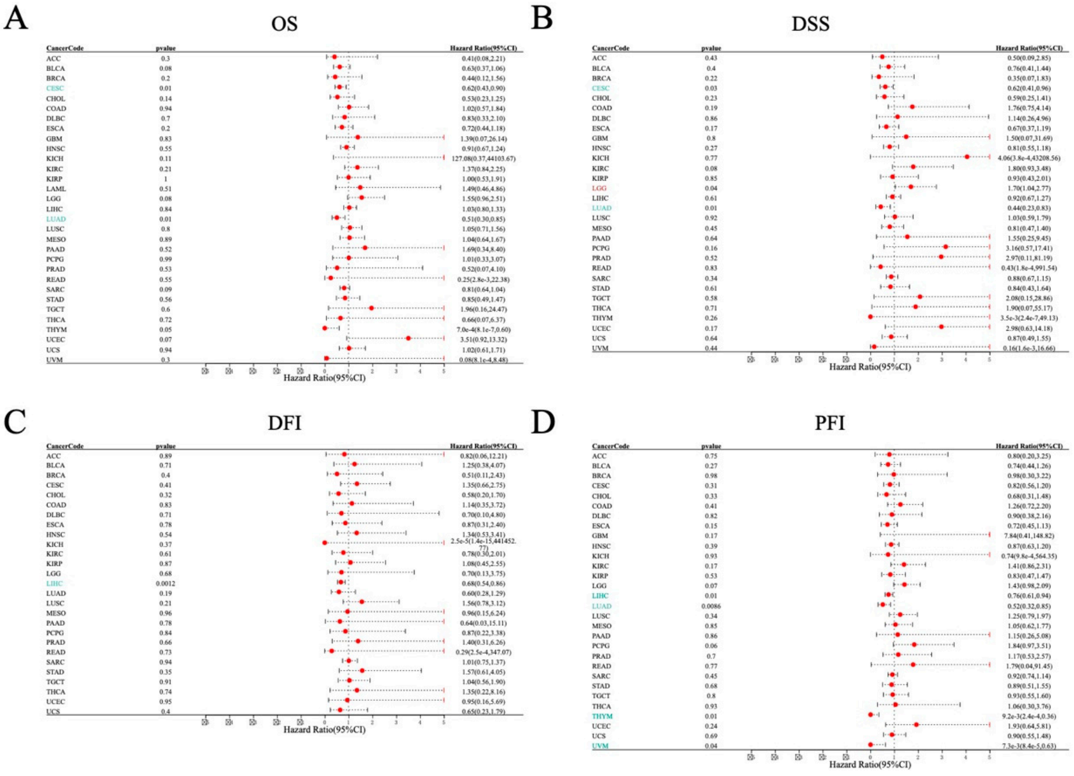

Supplementary Figure S5. Forest plot displays the survival of ISCA1 methylation expression levels in pan cancer.

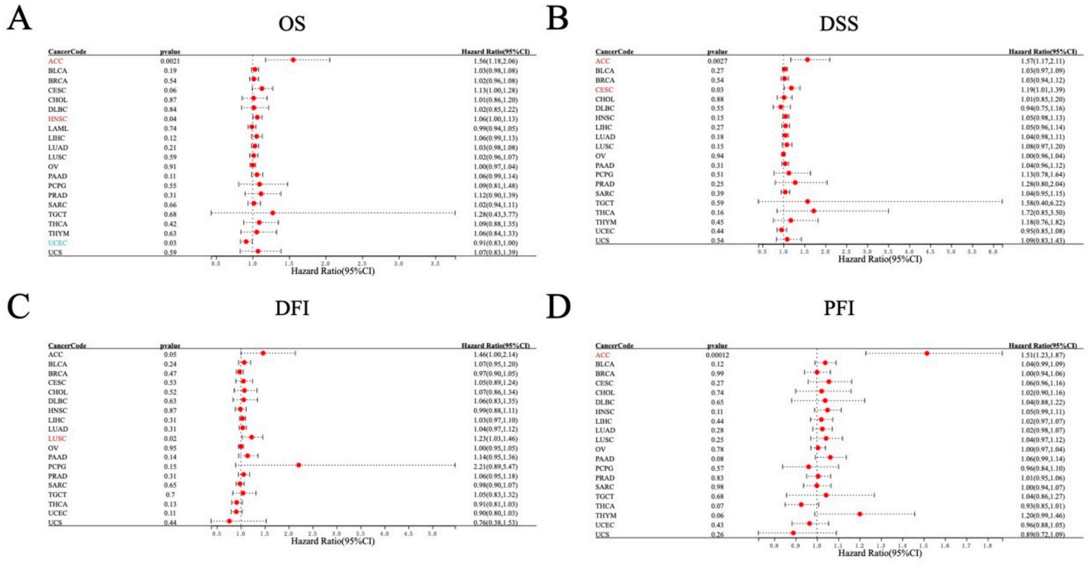

Supplementary Figure S6. Forest plot displays the survival of ENST00000311534 RNA expression levels in pan cancer.

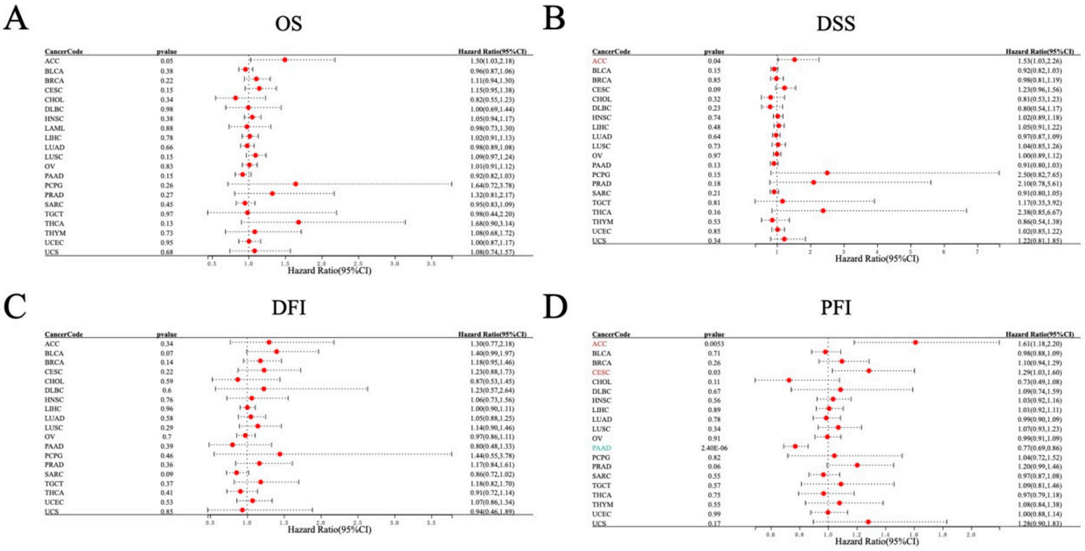

Supplementary Figure S7. Forest plot displays the survival of ENST00000326094 RNA expression levels in pan cancer.

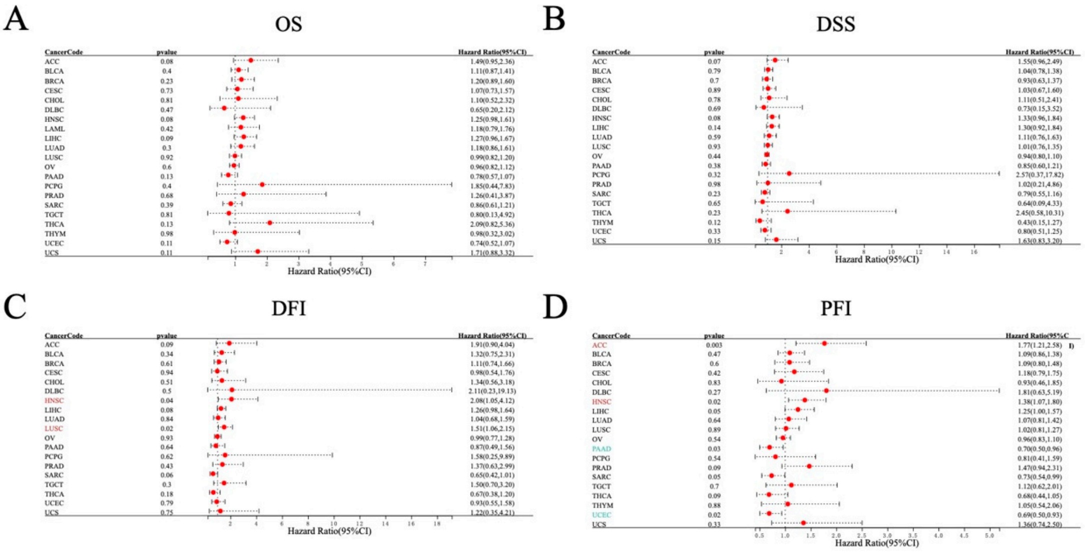

Supplementary Figure S8. Forest plot displays the survival of ENST00000375991 RNA expression levels in pan cancer.

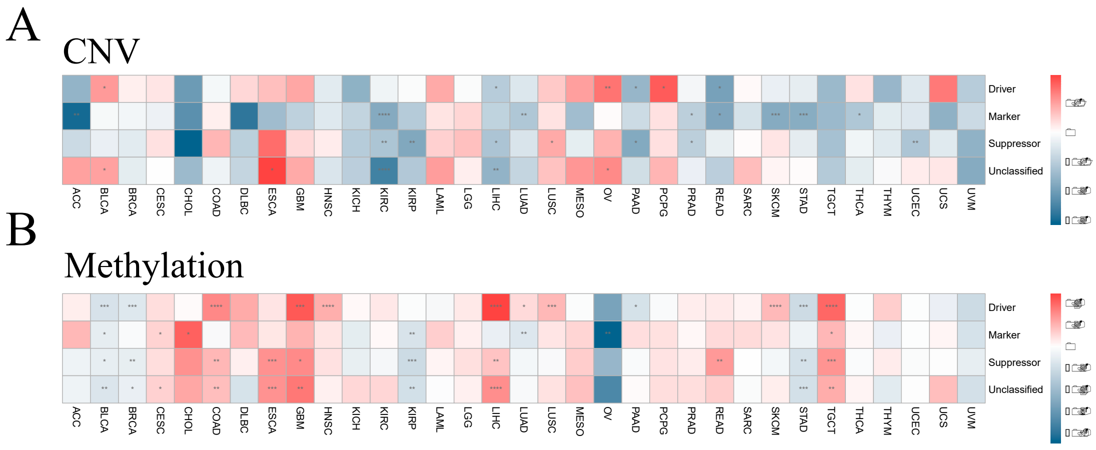

Supplementary Figure S9. The heatmap illustrates the correlations between (A) ISCA1 CNV, (B) ISCA1 methylation levels and ferroptosis-related gene set scores (*: $p < 0.05$, **: $p < 0.01$, ***: $p < 0.001$, ****: $p < 0.0001$).

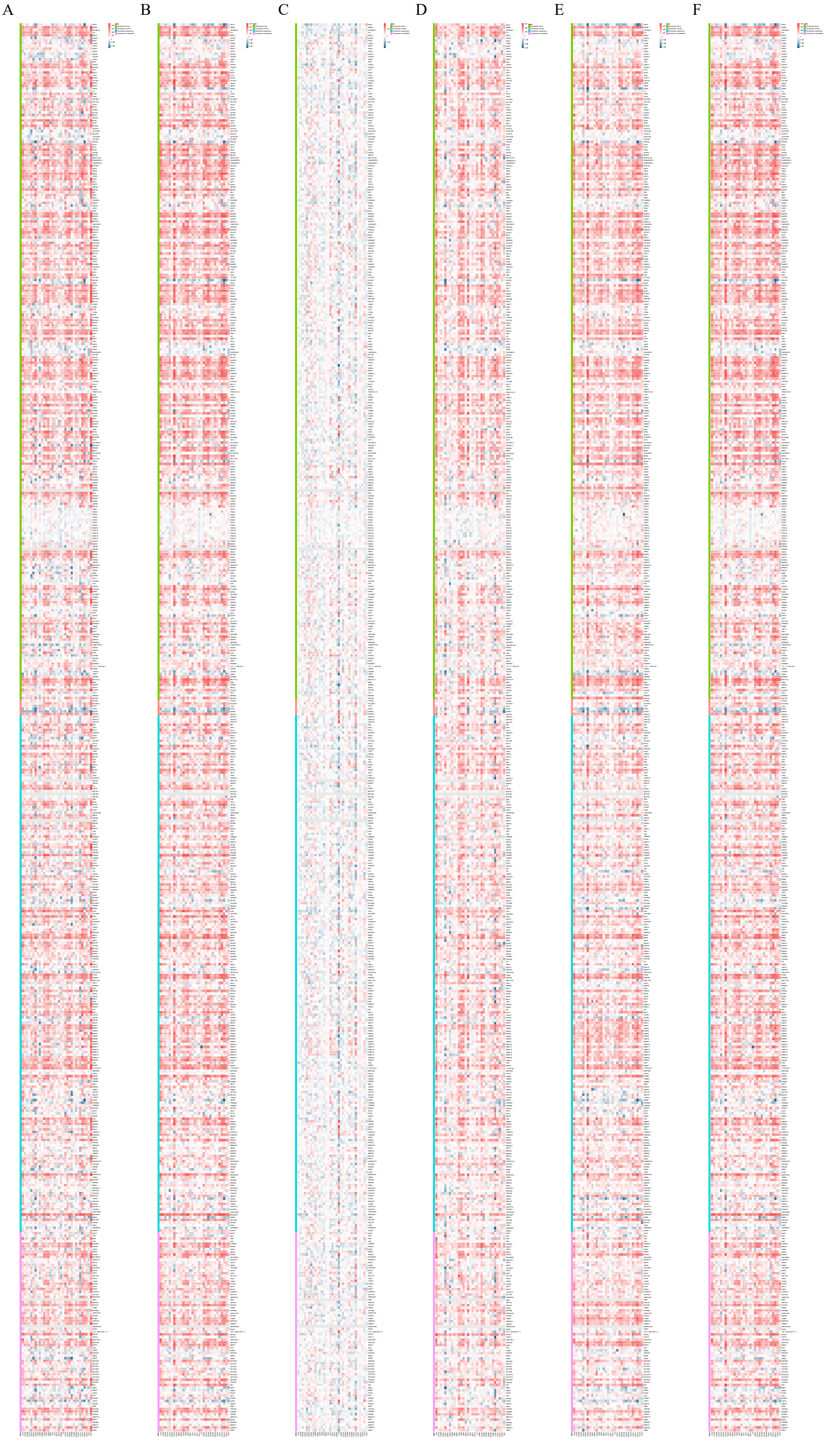

Supplementary Figure S10. Multi-omics analysis of *ISCA1* and all ferroptosis-related genes in various cancers. Heatmap showing the correlation analysis of (A) *ISCA1* RNA expression, (B) copy number variation (CNV) levels of *ISCA1*, (C) *ISCA1* methylation levels, (D) ENST00000311534 RNA expression, (E) ENST00000326094 RNA expression, (F) ENST00000375991 RNA expression and the RNA expression levels of ferroptosis-related genes (FRGs) (*: p < 0.05, **: p < 0.01, ***: p < 0.001, ****: p < 0.0001).

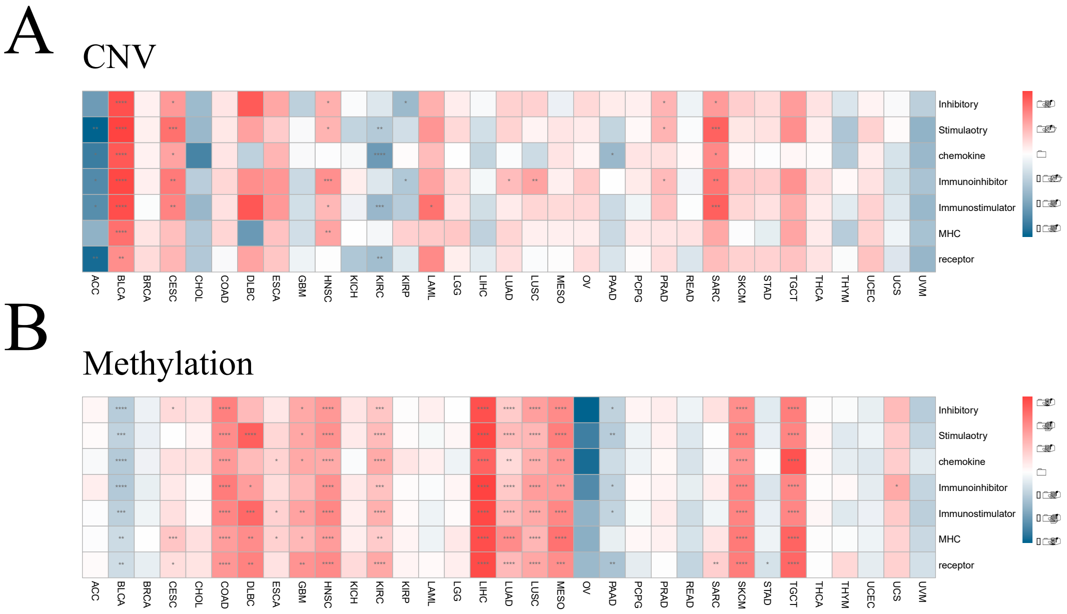

Supplementary Figure S11. The heatmap illustrates the correlations between (A) ISCA1 CNV, (B) ISCA1 methylation levels and immunoregulatory-related gene set scores (*: p < 0.05, **: p < 0.01, ***: p < 0.001, ****: p < 0.0001).

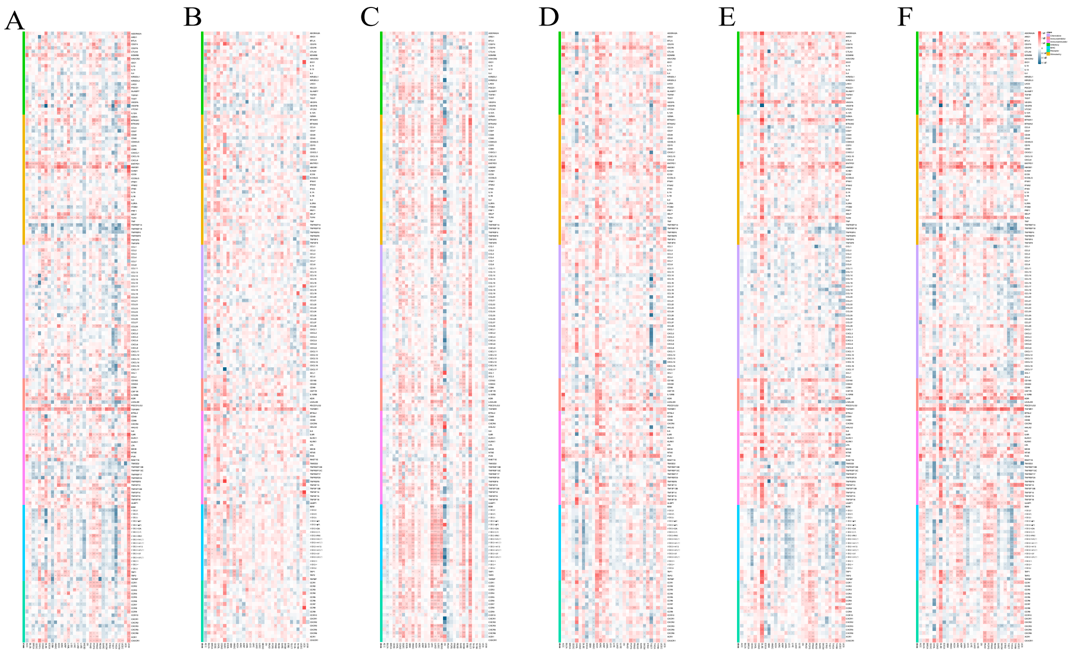

Supplementary Figure S12. Multi-omics analysis of *ISCA1* and immunoregulatory genes in each cancer. Heatmap showing the correlation between (A) *ISCA1* RNA expression, (B) copy number variation (CNV) levels of *ISCA1*, (C) *ISCA1* methylation levels, (D) ENST00000311534 RNA expression, (E) ENST00000326094 RNA expression, (F) ENST00000375991 RNA expression and the RNA expression levels of immunoregulatory genes (*: $p < 0.05$, **: $p < 0.01$, ***: $p < 0.001$, ****: $p < 0.0001$).

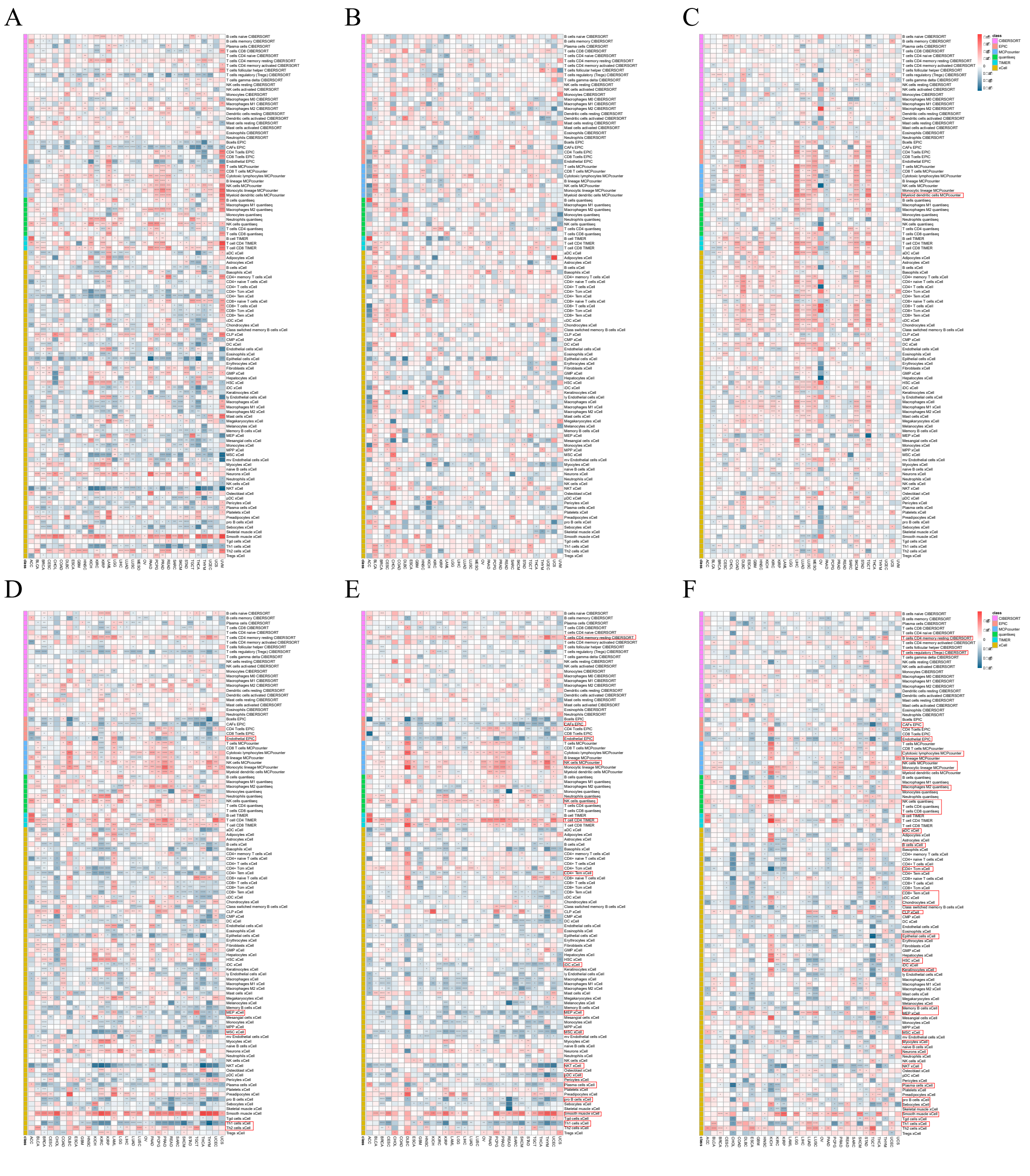

Supplementary Figure S13. The correlation of *ISCA1* with pan-cancer immune infiltrating cells. Heatmap depicting the correlation between (A) *ISCA1* RNA expression, (B) copy number variation (CNV) levels of *ISCA1*, (C) *ISCA1* methylation levels, (D) ENST00000311534 RNA expression, (E) ENST00000326094 RNA expression, (F) ENST00000375991 RNA expression and the infiltrations of all immune cells by six algorithms (*: $p < 0.05$, **: $p < 0.01$, ***: $p < 0.001$, ****: $p < 0.0001$).

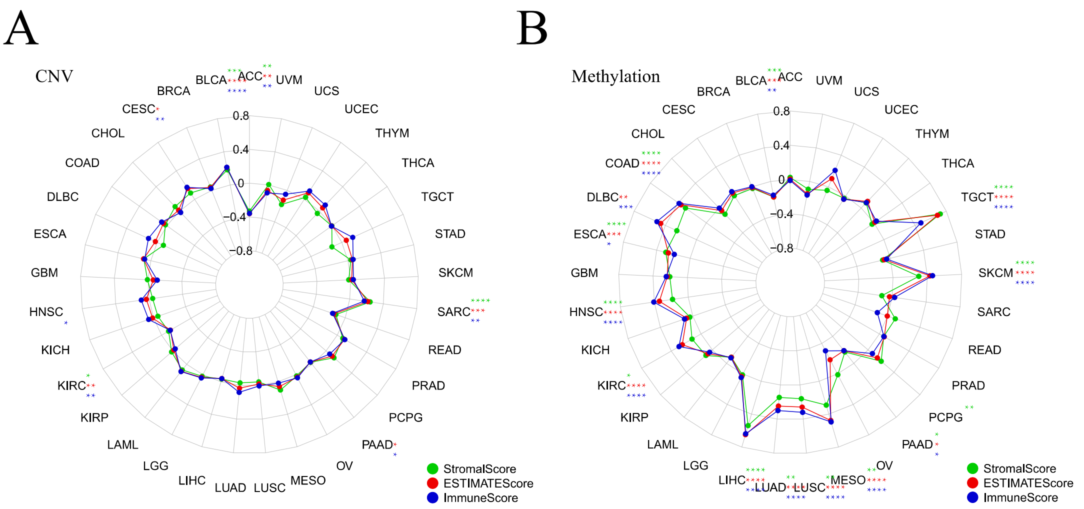

Supplementary Figure S14. Correlation analysis between (A) ISCA1 CNV, (B) ISCA1 methylation levels and three immune infiltration scores (*: p < 0.05, **: p < 0.01, ***: p < 0.001, ****: p < 0.0001).

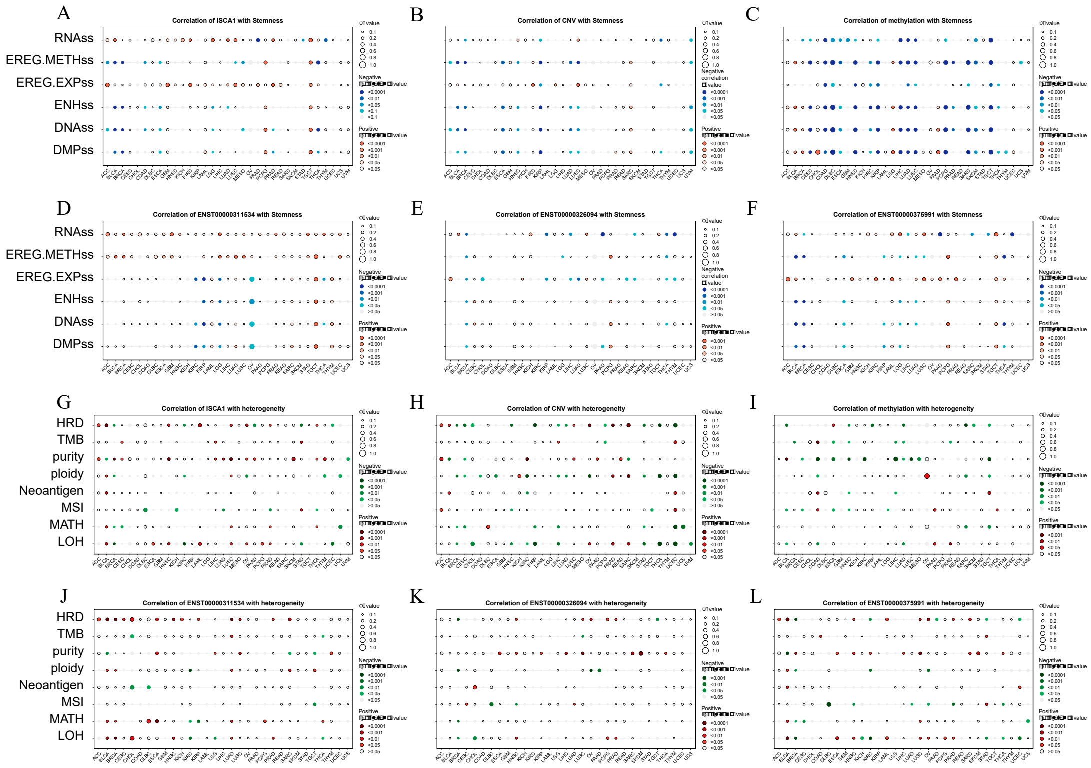

Supplementary Figure S15. Correlation Analysis between ISCA1 and tumor stemness, genomic heterogeneity in each cancer. (A-C) Bubble plots illustrating the correlation between RNA expression, CNV, as well as methylation of ISCA1 and tumor stemness. **(D-F)** Bubble plots illustrating the correlation between three transcripts of ISCA1 (ENST00000311534,

ENST00000326094, ENST00000375991) expression levels and tumor stemness. **(G-I)** Bubble plots illustrating the correlation between RNA expression, CNV, as well as methylation of ISCA1 and genomic heterogeneity. **(G-I)** Bubble plots illustrating the correlation between three transcripts of ISCA1 (ENST00000311534, ENST00000326094, ENST00000375991) expression levels and genomic heterogeneity. (*: $p < 0.05$, **: $p < 0.01$, ***: $p < 0.001$, ****: $p < 0.0001$. Bubble size represents the strength of the correlation, and bubble color indicates significance, with red indicating positive correlation and green indicating negative correlation).

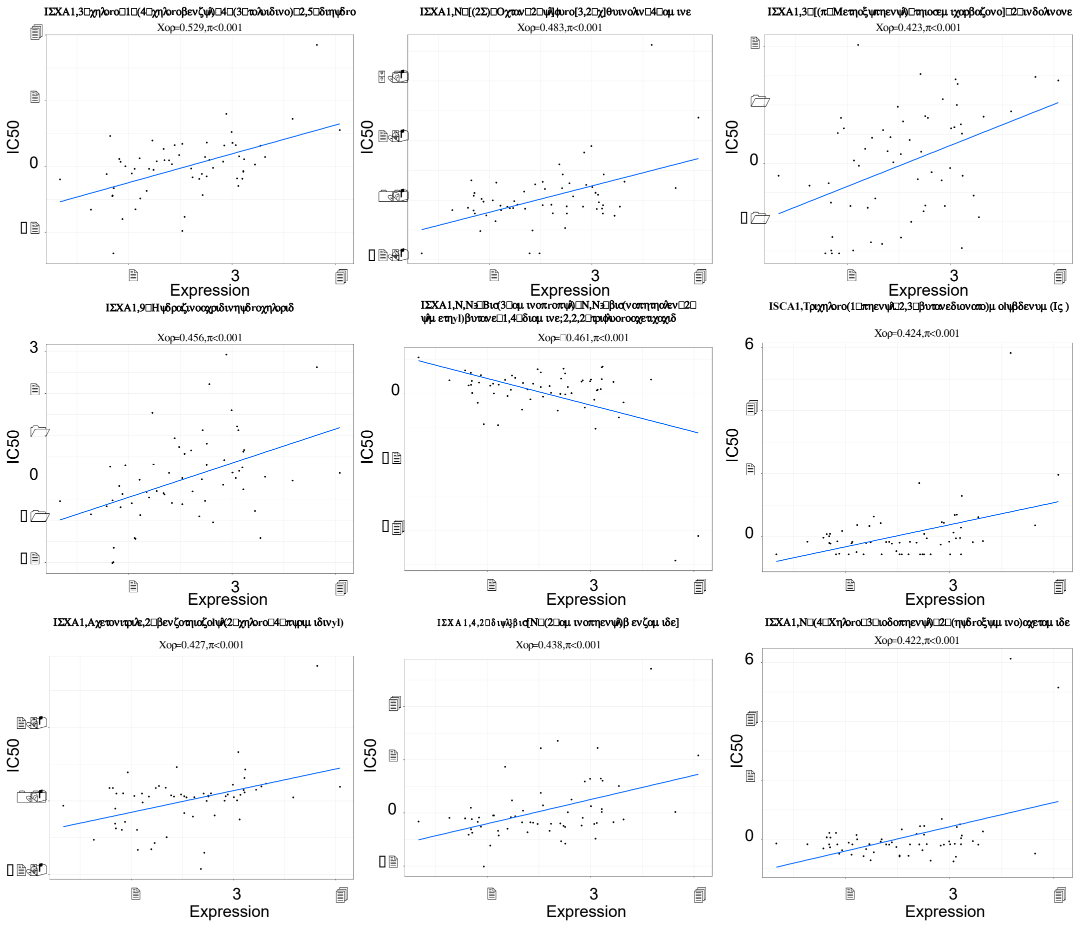

Supplementary Figure S16. The Scatter plots illustrating the correlation between *ISCA1* expression and the sensitivity of predicted drugs.

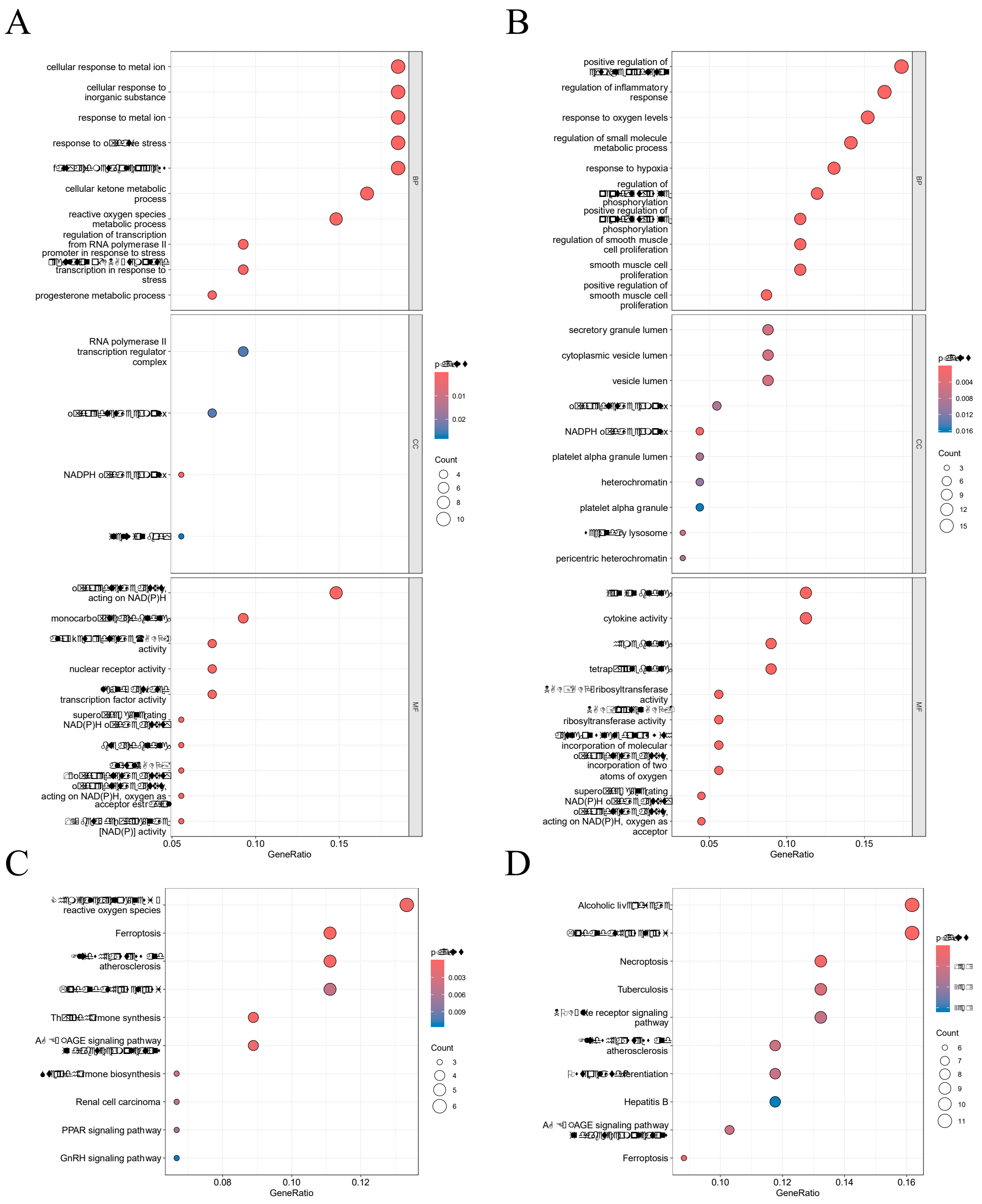

Supplementary Figure S17. (A-B) The GO analysis and (C-D) KEGG analysis of (A,C) up-expressed and (B,D) down-expressed ferroptosis-related genes between ISCA1-high and ISCA1-low tumors in THCA.

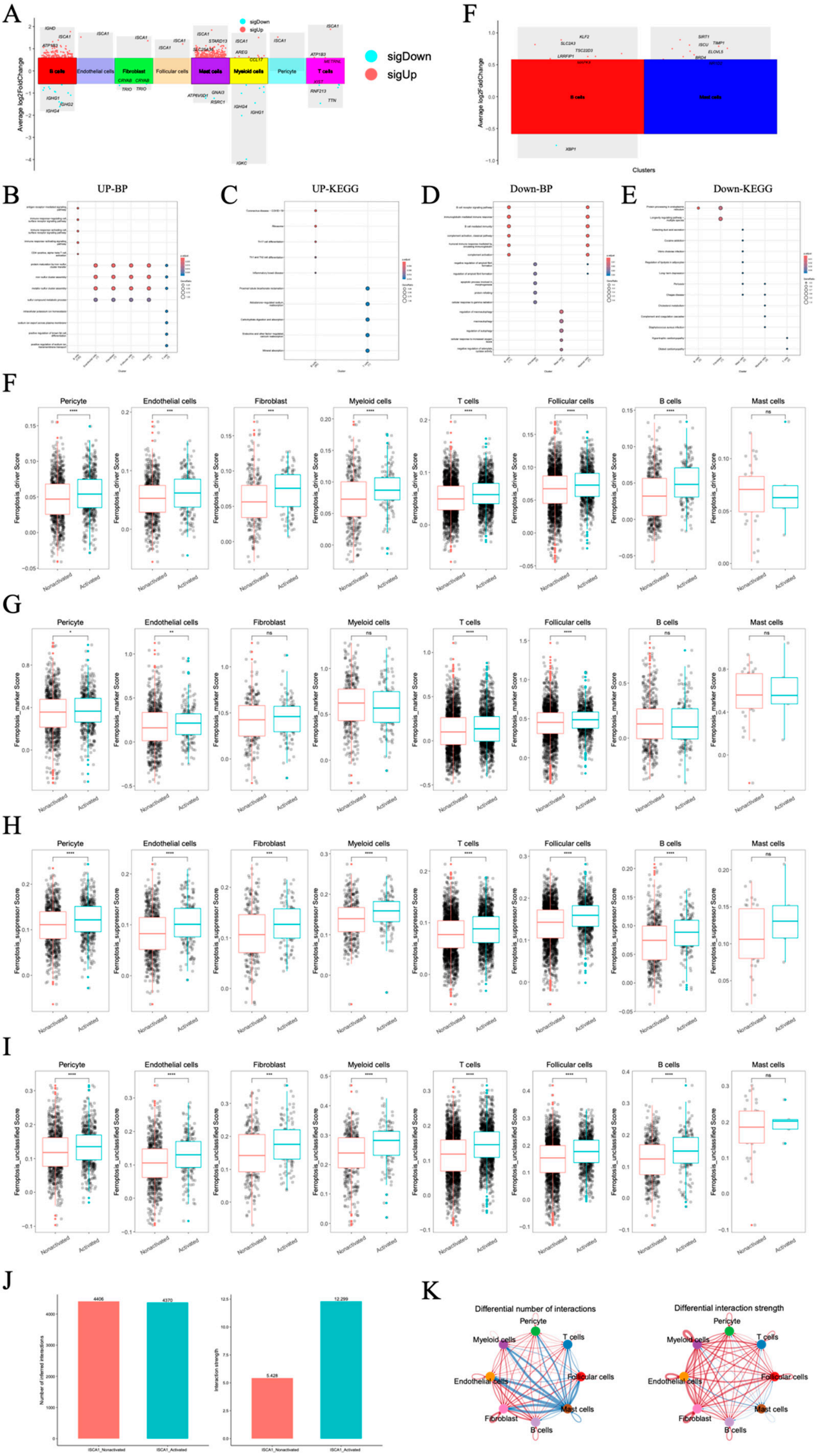

A
sigDown
sigUp
Average log2FoldChange
B cells
Endothelial cells
Fibroblast
Follicular cells
Mast cells
Myeloid cells
Pericyte
T cells
F
Clusters
B
UP-BP
C
UP-KEGG
D
Down-BP
E
Down-KEGG
F
G
H
I
Pericyte
Endothelial cells
Fibroblast
Myeloid cells
T cells
Follicular cells
B cells
Mast cells
Ferroptosis_driver Score
Ferroptosis_marker Score
Ferroptosis_suppressor Score
Ferroptosis_unclassified Score
Nonactivated
Activated
J
4406
4370
5.428
12.299
ISCA1_Nonactivated
ISCA1_Activated
K
Differential number of interactions
Differential interaction strength

Supplementary Figure S18. (A) The differential expression genes (DEGs) between ISCA1-activated and ISCA1-nonactivated in each cell type. The GO analysis of (B) up-regulated and (C) down-regulated genes in ISCA1-activated group in each cell type. The KEGG analysis of (D) up-regulated and (E) down-regulated genes in ISCA1-activated group in each cell type. (F) The differentially expressed FRGs between activated and nonactivated cells in each cell type. Compared the different scores of (G) ferroptosis-driver genes, (H) ferroptosis-suppressor genes, (I) ferroptosis-marker genes, (J) ferroptosis-unclassified regulator genes between activated and nonactivated group in each cell type. (K) The number of inferred interactions and interaction strength between cell types in ISCA1-activated and ISCA1-nonactivated group respectively. (L) The differential number of interactions and differential interaction strength between cell types in ISCA1-activated and ISCA1-nonactivated group respectively (*: $p < 0.05$, **: $p < 0.01$, ***: $p < 0.001$, ****: $p < 0.0001$).

Supplementary Table S1. The correlation coefficients and p values of the correlations between *ISCA1* RNA expression and predicted drugs.

Supplementary Table S2. The DEGs between ISCA1-high and ISCA1-low tumors in THCA.

Supplementary Table S3. The GO pathways of up-regulated genes in ISCA1-high tumors.

Supplementary Table S4. The GO pathways of down-regulated genes in ISCA1-high tumors.

Supplementary Table S5. The KEGG pathways of up-regulated genes in ISCA1-high tumors.

Supplementary Table S6. The KEGG pathways of down-regulated genes in ISCA1-high tumors.

Supplementary Table S7. The DEGs between ISCA1-activated and ISCA1-nonactivated cells of each cell type in THCA.

Supplementary Table S8. The GO pathways of up-regulated genes in ISCA1-activated tumors in each cell type.

Supplementary Table S9. The GO pathways of down-regulated genes in ISCA1-activated tumors in each cell type.

Supplementary Table S10. The KEGG pathways of up-regulated genes in ISCA1-

activated tumors in each cell type.

Supplementary Table S11. The KEGG pathways of down-regulated genes in ISCA1- activated tumors in each cell type.

Supplementary Table S12. The differentially-expressed FRGs between ISCA1-activate and ISCA1-nonactivated cells of each cell type in THCA.

Supplementary Table S13. The correlation coefficients and p-values between RNA expression, CNV and methylation levels of *ISCA1* and RNA expression levels of FRGs.

Supplementary Table S14. The correlation coefficients and p-values between RNA expression, CNV and methylation levels of *ISCA1* and RNA expression levels of immune-related genes.

Supplementary Table S15. The correlation coefficients and p-values between RNA expression, CNV and methylation levels of *ISCA1* and immune cell infiltrations.

Supplementary Table S16: The networks of ISCA1, FRGs, and immunoregulation genes in high-grade tumors.

Supplementary Table S17: The networks of ISCA1, FRGs, and immunoregulation genes in low-grade tumors.
